# Sustainable Adoption of Digital Health Innovations: Perspectives From a Stakeholder Workshop

**DOI:** 10.2196/11922

**Published:** 2019-03-25

**Authors:** Michelle Helena Van Velthoven, Carlos Cordon

**Affiliations:** 1 Department of Paediatrics University of Oxford Oxford United Kingdom; 2 International Institute for Management Development Business School Lausanne Switzerland

**Keywords:** adoption, digital health, stakeholders, telemedicine

## Abstract

**Background:**

There are various complex reasons that influence sustainable adoption of innovations in health care systems. Low adoption can be caused by a lack of support from one or more stakeholders because their needs and expectations are not always considered or aligned.

**Objective:**

This study aimed to identify stakeholders’ perceptions of barriers and facilitators toward the sustainable adoption of digital health innovations.

**Methods:**

A stakeholder workshop was attended by 12 participants with a range of backgrounds on August 25, 2017, including people representing the views from patients, carers, local hospitals, pharmacy retailers, health insurers, health services researchers, engineers, and technology and pharmaceutical companies in Switzerland. On the basis of adoption of innovation frameworks, we asked participants to interview each other about 3 factors influencing the adoption of digitally delivered health interventions: (1) Facilitators and barriers in the external system, (2) Needs and expectations of stakeholders, and (3) Safety, quality, and usability of innovations. The worksheets and videos generated from the workshop were qualitatively analyzed and summarized.

**Results:**

Facilitators for adoption mentioned were high levels of income and education, and digital health is a high priority to stakeholders. Main common interests of different stakeholders were patient satisfaction and job protection. Health care spending was a misaligned interest: although some stakeholders were keen on spending more to obtain or provide the highest quality of care, others were focused on reducing health care spending to provide cost-effective services. Switzerland’s diversity and complexity, in terms of its organization with 26 cantons (administrative divisions), were barriers as these made it harder to ensure interoperability of interventions. A culture of innovation was considered a push factor, but adoption was inhibited by persistent paper-based systems, a fear of change, and unwillingness to share data. The sustainability of interventions can be promoted by making them patient-centered, meaning that patients should be involved throughout their development.

**Conclusions:**

Promoting sustainable adoption of digital health remains challenging despite various push factors being in place. Barriers related to fragmentation, patient-centeredness, data security, privacy, trust, and job security need to be addressed. A strength is that people from a wide range of backgrounds attended the workshop. A limitation is that the findings are focused on the macro level. In-depth case studies of specific issues need to be conducted in different settings.

## Introduction

### Background

There is an ongoing discussion of how digital innovation can be used to improve health systems (the organization of people, institutions, and resources that deliver health care services to meet the health needs of people) around the world. Some countries have successfully adopted digital health innovation, for example, patient portals for Web access to clinicians are widespread in the United States [1]. However, health systems are notorious for slow and unsuccessful adoption of digital health innovation [2]. Currently, relatively few digital health innovations have been efficiently used despite various actors having expressed enthusiasm for digital health, and large investments are being made [3]. There are promising digital health innovations that are not widely adopted, for example, in the United Kingdom, general practice emails [4] or outpatient video consultations [5] are still not commonly used, or they are abandoned when people fail to scale them up or sustain use over time at an organization or system level. For example, a personal electronic health (eHealth) records program implemented in the United Kingdom between 2007 and 2010 was discontinued because very few people opened an account. Those who did, found it not easy or useful to use, and their expectations regarding self-management were not met [6].

Digital health innovations, also known as digitally delivered interventions, can be categorized in ways in which digital and mobile technologies are being used to support health system needs, including interventions for the public and patients, health care workers, health system managers, and data services [7]. Digital health innovations involve interactions within the health system, as well as the wider social, legal, political, and economic context [8]. There are numerous frameworks, theories, and systematic reviews of studies on the adoption of information and technology in health care [6,9-13]. This research has shown that not enough is known about the social, organizational, and cultural elements of successful implementation and adoption of digital health. Recently, a comprehensive framework on the diffusion of innovation in service organizations researched the spread, and sustainability of innovation in health services was expanded to research issues beyond adoption [14]. Factors influencing nonadoption and abandonment are complex and include the health condition, technology, value proposition, adopter system (comprising professional staff, patient, and lay caregivers), organizations, wider context, and interaction and mutual adaptation among all these factors over time. For example, more specific mentioned reasons for nonuse are complexities related to regulation [15,16], dealing with changes in workflow [17], the trust of the population regarding the privacy and security of data, and the conflicting interest of different stakeholders in the health care system [18,19].

### Objectives

One of the reasons for low adoption of innovations is a lack of support from 1 or more health care stakeholders, such as hospitals, health insurers, pharmaceutical companies, retailers, regulators, and patients [2]. The needs and expectations of different stakeholders are not always considered or aligned, and in some cases, there are conflicts among stakeholders. The aim of the workshop was to identify stakeholders’ perceptions as facilitators and barriers toward the successful adoption of digital health innovations, using Switzerland as a case study.

## Methods

### Setting

This stakeholder workshop took place on August 25, 2017 at the International Institute for Management Development (IMD) Business School in Lausanne, Switzerland. In this qualitative study, participants took part in small group exercises where examples from the Swiss health care system were discussed using relevant frameworks.

### Participants

Participants were selected through personal connections by searching the internet for relevant contacts and snowballing (eg, asking participants whether they know another relevant person). A total of 12 participants with a wide range of backgrounds attended the workshop, including people representing the views from patients, carers, local hospitals, pharmacy retailers, health insurers, health services researchers, engineers, and technology and pharmaceutical companies (Table 1). All participants provided verbal permission for the information they gave during the day to be used for research purposes. They were neither asked to pay for the workshop nor did they receive payments. Refreshments and lunch were provided during the day.

### Questions

On the basis of the nonadoption, abandonment, spread, scale-up, and sustainability framework for digital health innovations [14], we focused on 3 aspects from this framework: (1) Facilitators and barriers in the wider external system (political, regulatory, professional, and sociocultural), (2) Needs and expectations of stakeholders (eg, patients and the public, health professionals, health clinics/hospitals, pharmaceuticals, and insurers), and (3) development of safe, high-quality, and usable digital health innovations (technology). This framework and these aspects were chosen as they provide insights into the most recent and comprehensive thinking on the adoption of digital health innovations.

**Table 1:** Workshop participants’ background.

Participant number	Stakeholder and position, and background
1	University hospital: medical director
2	University hospital: head of electronic health domain
3	Patient representative: medical writer background
4	Patient representative: insurance consultant background
5	Patient representative: caretaker background
6	Academic: health sciences researcher
7	Pharmaceutical company: consumer health director
8	Pharmaceutical/technological company: computer engineer lead
9	Private equity: health investor specialist
10	Health insurance company: senior project manager
11	Pharmaceutical retail: specialist
12	Business: digital transformation specialist

### Exercise

We asked participants to take part in a “Round Robin exercise” in which they interviewed each other on 3 questions (see Multimedia Appendix 1). This method was chosen because it facilitates participants who are less assertive to contribute to a discussion by incorporating the responses of all participants to questions. Furthermore, participants are asked to provide answers without having listened to other participants, which reduces participants being influenced by others. This helps to overcome the issue that 1 or a few of the most talkative and dominant participants set the tone for the discussion, which might result in overlooking the ideas from others. We provided participants with detailed instructions on the exercise. A total of 3 groups of 4 participants were formed and given separate rooms. We provided each group 1 of the 3 questions to discuss (participants in group 1, question 1, etc). To ensure that all participants understood the exercise, they were given 15 min to discuss their question and the process of the exercise in their group and feed their understanding back to the facilitators. A total of 2 participants of each group rotated among the groups. During these rounds, 1 participant interviewed a member of another group (interviewee). After a 10-min interview, the participants reversed roles, and the interviewee became an interviewer. After the interview rounds, participants gathered in their own group to discuss and summarize their findings. Afterwards, all participants returned to the large room where each group presented their findings, and in-depth discussions explored their views on facilitators and barriers toward the adoption of digital health innovations.

### Data Collection and Analysis

We asked participants to write out their findings on large A4 worksheets. An assistant filmed the general discussion at the end of the workshop and took photos of the A4 sheets. The authors of this study were the moderators and observers of the workshop. They greeted the participants individually as they arrived and provided a welcome and introduction talk at the start of the workshop. They observed how the participants engaged in the exercise, answered questions, and listened to comments. Thematic analysis was conducted by the authors through separately watching videos in an active way (searching for meaning) to obtain an overview of the findings for each question and note their thoughts. Initial codes were given to findings (units of texts). The authors compared and discussed their coding, searched for themes, and sorted codes into potential themes. The authors carried out this process independently and discussed and compared their findings. Themes were related to each other to develop an explanation in relation to the research question. Close attention was paid to how the general discussion linked to the A4 sheets with findings. Data were summarized, and participants were sent the results and asked for feedback that was incorporated in the results.

## Results

### Overview

In the context of Switzerland, we describe 3 aspects of adoption of digital health innovations: (1) Facilitators and barriers in the wider external system (political, regulatory, professional, and sociocultural), (2) Interests of stakeholders (eg, patients and the public, health professionals, health clinics/hospitals, pharmaceuticals, and insurers), and (3) Development of safe, high-quality, and usable digital health innovations (technology).

### Key Facilitators and Barriers for Digital Health Adoption

Facilitators for digital health adoption mentioned were Switzerland being a rich country with high levels of education and digital health being a high priority to different stakeholders (Table 2). Diversity and complexity, particularly in terms of Switzerland’s organization with 26 cantons, were barriers as this was thought to make it harder to ensure interoperability of interventions. An innovation culture was seen as beneficial, but at the same time, it was thought that Swiss people feared change and were not willing to share data, and there is a tradition of paper-based systems in the country. A key success factor for digital health adoption involved clarification of the context around interventions, such as regulation for security and privacy. 

**Table 2:** Key facilitators, barriers, and success factors for digital health adoption.

Facilitators	Barriers	Success factors
Priority: Digital health is a priority to improve the quality of health services in Switzerland; politicians are educated on the importance and issues related to digitization	Decentralization and lack of interoperability: Switzerland has 26 cantons and different health laws; there is no common interface for electronic patient record interoperability. Furthermore, there are 4 different languages used (French, German, Italian, and Romansh)	Context clarification: There is a need to put national regulation in place for security, privacy, and “hacking” of digital health innovations (medical devices)
High-income country: Large investments are being made in innovation; furthermore, most people are educated and have personal technologies such as smartphones	Lack of long-term planning: People can change health insurance every year, which limits long-term investment of insurance companies	Responsibilities clarification: The responsibilities of different stakeholders need to be clarified, for example, who pays which costs
Size: Switzerland is a small country where people know each other	Experience: There is relatively limited experience within Switzerland	Import: Switzerland could make better use of the considerable number of expats bringing in their expertise
Culture of innovation: Switzerland has a thriving start-up, and health companies’ “valley” and local initiatives are trialed. Many initiatives over the past two decades have taken place, including the establishment of pharma companies	High health care costs: The overuse of health care is insufficiently limited. Patients pay high insurance and in return want high-quality services. There is a lack of willingness to pay for digital health	Value proposition clarification: The benefits and impact on costs for citizens, for example, fees for services, need to be clarified
Consensus: Swiss people like to solve problems together with all stakeholders and common agreement. Opportunities to share data are provided	Low agility: There is a general lack of willingness to share personal data. Rigid and slow adoption of innovation, for example, a paper billing system (“System de Tarification”)	Change enablement: Change processes need to be better enabled to achieve an appropriate proportion of people using a digital health innovation

Furthermore, the benefits and costs for patients and the public would have to be better explained.

### Aligned and Differing Interests of Stakeholders

The main common interest for different stakeholders (see Table 3) was increased efficiency, which could lead to a reduction in cost. It was mentioned that these savings could then be used, for example, for research and development of drugs for rare diseases. Conflicts were mentioned for health care spending, although some stakeholders such as patient groups and certain companies were keen on spending more to obtain the highest-quality care, and in the long-term, this could make health care spending unsustainable. Furthermore, there were conflicting interests mentioned for the better use of data, for example, although patients would be interested in contributing to research and development of drugs and devices, they were concerned about threats to their privacy.

### Facilitators and Barriers Toward Developing Digital Health Interventions

To develop safe, high-quality, and usable innovations, it was mentioned that agile engineer approaches could be used to more rapidly develop innovations with measurable outcomes, but traditional health care companies such as pharmaceutical companies are more used to lengthy drug development processes (see Table 4). Patient-facing interventions should be patient-centered, meaning that patients should be involved in their development. However, there was said to be a lack of studies on patient involvement in digital health studies. Data derived from digital health tools could be used for individualization of health care if personal data were to be obtained. Although more jobs are needed to enable digitization in health care, there were concerns about the loss of jobs caused by digitization.

**Table 3:** Aligned and conflicting interests of stakeholders.

Stakeholders, aligned interests		Conflicting interests
**Pharmaceutical companies**		
	The possibility to access more targeted data to be used in the development of drugs	The possibility of being left behind when data are not shared
	Potentially increased customer satisfaction	Patients could become more demanding, which could result in the need to change culture and processes
	Increased efficiency in the use of resources, for example, by increased compliance of patients taking drugs	A potential reduction in drug sales, for example, by paying per pill versus paying per box of pills, which improves the accuracy of drugs dispensing
	Increase in rare disease research and development on the basis of gains from other disease areas	Costs could be escalating by investment in digital health with uncertain returns
**Hospitals and health workers**		
	Saving staff time and lowering costs	Uncertainty about who pays for what time
	Better collaboration with colleagues, for example, by opportunities for information sharing	Health workers could be losing some of their autonomy, for example, by more traceable work by tracking what a staff member is doing
**Patients and the public**		
	The opportunity to contribute feedback to research and development, for example, postmarket feedback on adverse effects of drugs	Threats to privacy, for example, it being uncertain what happens to personal data
**Insurers**		
	An increased efficiency in treatment, for example, reduced time in the hospital	Challenges for balancing expensive treatment versus the cost to society. It might be the right decision to pay for expensive treatment if the patient can return to society sooner
	Expert high-quality care for patients	Private versus mandatory insurance, for example, risk selection on the basis of available personal data. Even though legally this is not possible, it is happening unofficially

**Table 4:** Facilitators and barriers toward developing safe, high-quality, and usability innovations.

Facilitators	Barriers
Agile approaches can be used to develop digital health innovations	Pharmaceutical companies are not used to using agile approaches
A sufficient mass of secure data, for example, use of data for individualization of treatment	People do not want to share data and are concerned about data ownership
The government is seen as credible and could use this to, for example, define what a medical device is and support the establishment of standards for safety	Companies developing digital health innovations are not always seen as credible
Patient-facing interventions should be focused on patients, which can be achieved by, for example, involving them throughout the development process	Lack of understanding what patient-centeredness really means through studies on patient involvement
Clinicians would like to be involved	Clinicians’ role for involvement needs to be defined
Interventions need to have shared benefits and measurable outcomes	There are concerns about how to measure cost-effectiveness
There is a need for more jobs in digital health	People are concerned about job losses
Insurers need to be transparent about their willingness to pay for digital health innovations	People are able to change their health care insurance

## Discussion

### Principal Findings

This paper reports on findings from a workshop with the aim to identify stakeholders’ perceptions of barriers and facilitators toward the successful adoption of digital health innovations. The workshop led to 3 main interesting insights. First, there was a lack of understanding of how the different stakeholders in the ecosystem work and how they are incentivized. For example, it was assumed that 1 of the key roles of health insurance is to control health care costs. However, the reality in Switzerland and other countries with mandatory health insurance is that there is neither incentive nor a role for insurers to control or reduce health care–related costs [20]. Second, there is a lack of patient involvement in the development of digital health initiatives. Despite the push for patient-centeredness [21], 2 representatives from patient organizations in our workshop explained that they were never consulted by any of the other stakeholders. Third, stakeholders launch initiatives with a lack of understanding of the basics of digital solutions. Usability was frequently mentioned as the main problem in digital solutions in health care even though there is a considerable amount of literature on developing user-centered and engaging interventions [22-24].

Topics that were expected to be discussed included issues around data security, as they are frequently discussed in popular media [25]. According to the World Health Organization (WHO), Switzerland adopted a national eHealth policy in 2007 and a policy on multilingualism in eHealth in 2010 [26]. Switzerland does not have a national policy or strategy on the use of social media by government organizations; health care organizations promote health messages as part of health promotion campaigns, and individuals use social media to learn about health issues. Furthermore, there is no policy or strategy for governing the use of data in the health sector or by private companies [26]. Putting these policies in place could help to clarify the context in which digital health innovations can be safely and securely used in Switzerland. Participants mentioned that Swiss people do not like to share data and that Switzerland is a “paper country.” The culture of change was not discussed even though there is a lot of academic literature on this topic [17,27], because participants assumed that in our connected world, people are open to digital innovation adoption. It was not expected that the risk of losing employment was an important issue in Switzerland given its high employment rate and difficulty with hiring specialized people.

### Strengths and Limitations

Strengths of this workshop include that we invited people from a wide range of backgrounds and we purposively selected a small number of people to allow for in-depth discussions. Limitations include that the workshop was only held for 1 day and that the findings are focused on the macrolevel and do not provide meso or microlevel insights. The findings are contextual to Switzerland and not necessarily generalizable to other settings.

### Comparison With Previous Work

Our findings have implications for policy makers, practitioners, and companies who want to develop digital health innovations. First, companies should keep in mind that countries such as Switzerland, despite being small, can be highly fragmented. Integration of care and data and scale-up of innovations are challenging with tiered governance and 26 cantons with different health laws. As a result, Switzerland does not have a national eHealth record system [26]. Furthermore, this means that many telemedicine services (eg, teleradiology, teledermatology, telepathology, and remote patient monitoring services) and mobile health programs (eg, toll-free emergency, health call centers, and community mobilization for accessing/providing health information) are regional, local, or in an information state [26]. Second, companies should consider the needs of users of digital health innovations and involve them throughout the design and development. Third, the incentives for digital health–related jobs should be better organized. Although Switzerland has a high employment rate and difficulty with hiring specialized people, job losses are a concern for Swiss people that limit implementation-readiness of providers. Although some jobs may be replaced by digital health innovations, this also creates new jobs. According to the WHO, less than 25% of health sciences students receive preservice training in eHealth, and more than 75% of health professionals receive in-service training in eHealth [26]. Educating and (re)training the workforce in digital health will be important to reduce job loss concerns.

### Conclusions

In conclusion, countries such as Switzerland with an advanced infrastructure for information and communication technology and a high quality of care make an attractive place for companies to develop digital health innovations. However, barriers related to fragmentation, patient-centeredness, trust, and job security need to be addressed.

## Appendix

Multimedia Appendix 1 Digital health ecosystem Round Robin exercise.

## Abbreviations

eHealth: electronic health
IMD: International Institute for Management Development
WHO: World Health Organization
