# Supplementary material for: Sustainable Adoption of Digital Health Innovations: Perspectives From a Stakeholder Workshop
Source: J Med Internet Res. 2019 Mar 25;21(3):e11922. doi: 10.2196/11922 (PMC6452285; doi:10.2196/11922)
Supplement: Multimedia Appendix 1 [file jmir_v21i3e11922_app1.pdf]

Digital Health Ecosystem

Round Robin Exercise

This process has four steps:

1. You will meet briefly with your group to discuss the question you will ask during the interviews you will conduct. Question assignments are given on page 3 while the questions are listed on page 2.
2. You will conduct several interviews (and be interviewed).
3. You will meet again with your group to summarize the responses, and nominate a spokesperson to present your findings.
4. Your group will present your findings for discussion.

Step 1

Your group will meet in an assigned room to clarify the assigned question

Step 2

Your task is to interview someone whom you do not know especially well by asking them your assigned question. Note the person you interview has a different question from the one you have been assigned. You should probe to make sure that the person is thinking freshly, but do not disagree or argue. You will have 10 minutes for your interview and will, in turn, be interviewed for about 10 minutes.

Please note: When 10 minutes have passed, you will be instructed to change roles with the interviewer becoming the interviewee. When 20 minutes have passed, you will be instructed to change partners, according to the rotation given on page 3. We will do this for two rounds of interviews.

Remember: Always ask the same question, but always answer a different question - and never your own.

Step 3

After the 2 rounds of interviews each group will prepare a summary and nominate one member to present the group's findings to all participants in this exercise.

Step 4

All groups will return to the main room. The group member nominated in Step 3 will present the findings of the group.

Digital Health Ecosystem

Round Robin Exercise – Discussion Questions

1. **Success factor 1: Key Facilitators and Barriers**
   What are the facilitators and barriers for adoption of digital health innovations in Switzerland? What would you consider a successful adoption of a digital health innovation?

2. **Success factor 2: Aligned Interests of Stakeholders in Switzerland**
   What are the areas where the interests of different stakeholders coincide and where they conflict in the digital health ecosystem? What are the benefits, costs and risks for digital health innovation for different stakeholders?

   (Stakeholders: Patients and consumers, Health professionals, Health clinics/hospitals, Academics, Pharmaceuticals, Insurers, Retailers, Large generic technology companies, Medical device companies, Start-ups, Investors, Venture Capitalists etc.)

3. **Success factor 3: High-quality innovations that work in practice**
   What is important for developing, evaluating and implementing a 'good' digital health intervention or innovation? What are the most important factors for a digital health innovation to be adopted?

Digital Health Ecosystem

Round Robin Exercise

Rotation Sequence

There are three questions:

Group 1 asks question #1
Group 2 asks question #2
Group 3 asks question #3
